# Optimizing Alveolar Ridge Preservation and GBR: A Systematic Review of Membranes, Grafts, and Soft Tissue Strategies for Personalized Care

**DOI:** 10.1155/ijod/3754780

**Published:** 2026-08-24

**Authors:** Angelo Michele Inchingolo, Grazia Marinelli, Pietro Lauria, Pierluigi Marotti, Silvia Chieppa, Francesco Inchingolo, Andrea Palermo, Massimo Del Fabbro, Alessio Danilo Inchingolo, Gianna Dipalma

**Affiliations:** ^1^ Department of Interdisciplinary Medicine, University of Bari “Aldo Moro”, Bari 70124, Italy, uniba.it; ^2^ Department of Biomedical, Surgical and Dental Sciences, Milano University, Milan 20122, Italy, unimi.it; ^3^ Department of Experimental Medicine, University of Salento, Lecce 73100, Italy, unisalento.it; ^4^ Unit of Maxillo-Facial Surgery and Dentistry, Fondazione IRCCS Ca’ Granda Ospedale Maggiore Policlinico, Milan 20122, Italy, policlinico.mi.it

**Keywords:** alveolar ridge preservation, bone graft materials, guided bone regeneration, implant stability, post-extraction resorption, regenerative protocols, resorbable membranes, soft tissue management, surgical timing

## Abstract

**Background:**

Alveolar bone loss following tooth extraction remains one of the main challenges in implant dentistry. To counteract the physiological resorption that occurs post‐extraction, regenerative techniques such as alveolar ridge preservation (ARP) and guided bone regeneration (GBR) have been developed. These approaches use bone graft materials and barrier membranes to preserve or restore alveolar architecture, facilitating optimal implant placement.

**Objective:**

This systematic review aims to critically evaluate the most relevant and methodologically robust clinical studies comparing the effectiveness of ARP and GBR techniques. Specific attention is given to membrane types, graft materials, surgical timing, and soft tissue management strategies, assessing their impact on biological, functional, and esthetic outcomes.

**Methods:**

Fourteen high‐quality randomized controlled trials were selected and analyzed. Key outcome measures included vertical and horizontal bone gain, implant survival rates, soft tissue stability, postoperative complications, and patient‐reported satisfaction.

**Results:**

Modern resorbable membranes demonstrate comparable bone regeneration outcomes to nonresorbable ones, while minimizing surgical invasiveness. Titanium mesh offers superior bone gain in complex defects but is associated with a higher complication rate. Early intervention may improve outcomes. The choice of graft material influences both osteogenic potential and structural stability. However, generalizability is limited by small sample sizes, short follow‐up periods, and exclusion of patients with systemic comorbidities.

**Conclusions:**

Successful alveolar regeneration requires a personalized treatment approach, considering defect morphology and individual patient characteristics. Future research should prioritize larger, long‐term, multicenter studies that include real‐world patient populations to support the development of more predictive and patient‐centered clinical protocols.

## 1. Introduction

### 1.1. Clinical Relevance of Alveolar Bone Loss

Alveolar bone loss following tooth extraction stands as a central clinical issue within the fields of implantology and regenerative oral surgery [1]. This biologically driven resorptive phenomenon occurs predictably in both vertical and horizontal dimensions, leading to a substantial reduction in the volume of bone available for future prosthetic rehabilitation [2, 3]. However, the repercussions of post‐extraction bone loss extend beyond mere quantitative loss. The structural integrity, spatial conformation, and qualitative characteristics of the alveolar ridge are also adversely affected [4–7]. These alterations may compromise the feasibility of proper implant placement, diminishing the predictability of achieving both primary mechanical stability at placement and long‐term secondary stability during osseointegration [8, 9]. As a result, untreated bone resorption may significantly impair functional rehabilitation, reducing masticatory efficiency and altering occlusal load distribution [10–13]. Moreover, from an esthetic standpoint, these changes can distort gingival symmetry and soft tissue contours, thereby compromising smile esthetics and overall patient satisfaction [14–16].

### 1.2. Necessity of Ridge Preservation and Regeneration

Given the aforementioned challenges, the preservation and regeneration of alveolar ridge architecture have become critical components in the planning of successful implant‐based oral rehabilitation [17–19]. A well‐preserved ridge not only facilitates ideal three‐dimensional implant positioning but also ensures the health and maintenance of surrounding peri‐implant soft tissues [20–24]. Therefore, strategies aimed at maintaining or reconstructing alveolar bone volume are increasingly viewed as essential prerequisites for obtaining long‐term success—both from a functional standpoint, such as chewing efficiency and phonetics, and from an esthetic perspective, including soft tissue support and gingival harmony [25–28]. These therapeutic goals underscore the need for proactive and biologically driven interventions immediately following tooth extraction.

### 1.3. Overview of ARP and GBR Techniques

**Figure 1:**
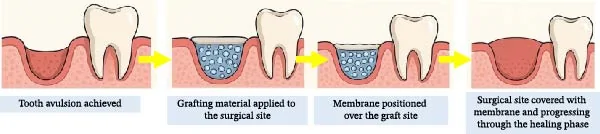
Healing process after bone regeneration.

In response to the complex biological cascade that follows tooth extraction, two primary regenerative strategies have emerged and evolved over the past decades: alveolar ridge preservation (ARP) and guided bone regeneration (GBR). These evidence‐based protocols are designed to mitigate the extent of post‐extraction bone loss by intervening early in the healing process [29–31]. Their core principle lies in the use of bone grafting materials—ranging from autogenous and allogenic to xenogenic and alloplastic origins—often in combination with physical barrier membranes. These membranes are essential for excluding soft tissue ingress, thereby creating a protected environment conducive to osteogenic cell proliferation and matrix deposition (Figure 1). Through this orchestrated combination of biological cues and mechanical stability, ARP and GBR serve to regenerate alveolar structures, re‐establishing favorable anatomical conditions for prosthetic rehabilitation via implant placement [32–35].

### 1.4. Advancements in Materials and Surgical Protocols

Recent scientific progress has substantially enhanced the clinical utility and outcomes of ARP and GBR. Notably, biomaterials have been developed with superior biocompatibility and enhanced osteoconductive and osteoinductive properties, enabling more predictable integration and regeneration [36–39]. In parallel, surgical techniques have evolved to become increasingly less invasive, minimizing patient discomfort and optimizing postoperative healing. These developments have broadened the range of viable clinical applications and facilitated the implementation of personalized regenerative protocols tailored to the anatomical, biological, and prosthetic needs of individual patients. Such innovations have transformed regenerative procedures from experimental solutions to standard components of modern implantology [40–42].

### 1.5. Clinical Decision‐Making Complexity

Despite the promising advances in materials science and surgical technique, the clinical application of ARP and GBR remains fraught with complexity. The clinician must make informed decisions regarding the choice between resorbable and nonresorbable membranes, the selection of an appropriate grafting material, the ideal timing for surgical intervention, and the optimal approach for managing the peri‐implant soft tissues.

### 1.6. The Need for a Critical and Systematic Review

The growing volume of literature addressing ARP and GBR reflects the clinical relevance and scientific interest in this domain. However, this body of evidence is often characterized by methodological heterogeneity, diverse clinical protocols, and sometimes contradictory results [43–45]. Such variability impedes the formulation of universally accepted clinical guidelines and calls for a thorough, critical reassessment of the existing data. A systematic review that emphasizes methodological rigor and clarity is necessary to distill reliable conclusions and guide clinical practice with greater precision. The integration of well‐designed clinical trials, longer follow‐up periods, and real‐world patient cohorts is essential to bridge the gap between academic research and everyday clinical reality [46–49].

### 1.7. Objectives of the Present Review

In light of these considerations, the present systematic review aims to provide a comprehensive and up‐to‐date evaluation of the most methodologically sound and clinically relevant studies on ARP and GBR [50–53]. Special emphasis will be placed on comparing the effectiveness of various graft materials and membrane types, evaluating the timing and sequencing of surgical interventions, and analyzing strategies for optimal soft‐tissue management. These elements are now widely acknowledged as fundamental to ensuring biological integration, functional restoration, and long‐term esthetic success [54–57]. Furthermore, the review will critically highlight common limitations encountered in the current literature—such as limited sample sizes, short observation periods, and the underrepresentation of patients with complex medical profiles—which currently hinder the generalizability of findings and represent key challenges for future research advancement in this essential field of oral rehabilitation [58–62].

## 2. Materials and Methods

### 2.1. Eligibility Criteria (PICO)

This systematic review was designed according to the PICO framework to define inclusion criteria:

• Population (P): Patients undergoing ARP or GBR following tooth extraction, including those planned for subsequent implant placement.
• Intervention (I): Application of different types of barrier membranes (resorbable collagen, nonresorbable, cross‐linked membranes), various graft materials (autografts, xenografts, allografts, and synthetic substitutes), and soft tissue management techniques (e.g., connective tissue grafts [CTGs] and flap designs).
• Comparison (C): Comparison among different membrane types, graft materials, soft tissue strategies, and timing of implant placement (immediate vs. delayed).
• Outcomes (O): Clinical and radiographic outcomes including bone volume preservation, dimensional stability, membrane exposure rates, soft tissue thickness, esthetic results, and implant success rates.

### 2.2. Research Question

The main research question addressed by this review is as follows:

*Which combinations of membranes*, *graft materials*, *and soft tissue management strategies provide the most effective and predictable outcomes in alveolar ridge preservation and guided bone regeneration to optimize implant therapy?*

This question guided the systematic identification and synthesis of clinical evidence to inform personalized regenerative protocols.

### 2.3. Information Sources and Search Strategy

A comprehensive literature search was conducted in May 2025 across three major electronic databases: PubMed, Scopus, and Web of Science. The search strategy was constructed using a combination of controlled vocabulary and free‐text terms related to the topic. Key terms included: “alveolar ridge preservation,” “guided bone regeneration,” “membrane,” “graft material,” “soft tissue management,” “implant placement,” and their synonyms. Boolean operators (AND and OR) were used to combine terms effectively.

The full search syntax was adapted for each database to maximize the retrieval sensitivity. Additionally, the reference lists of eligible articles and relevant reviews were manually screened to identify further pertinent studies.

### 2.4. Data Extraction

**Figure 2:**
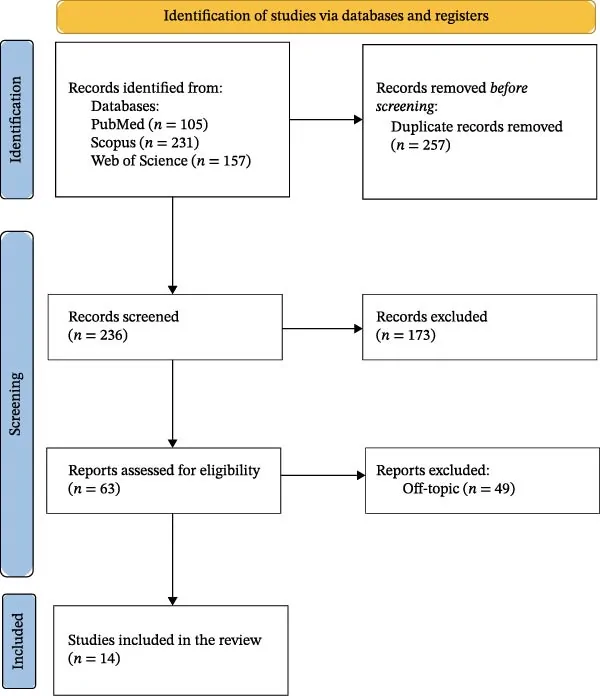
PRISMA flowchart.

Data extraction was conducted independently by the reviewers using a standardized data collection form. Extracted variables included the author and year, study design, sample size, patient demographics, types and characteristics of membranes and graft materials used, soft tissue management techniques, timing of implant placement, follow‐up duration, and reported clinical and radiographic outcomes. The extracted data were cross‐checked for accuracy and completeness before the analysis (Figure 2).

### 2.5. Quality Assessment

The quality of the included studies was carefully evaluated to determine the risk of bias across several domains, including selection, measurement, and reporting biases. Overall, most studies demonstrated a low‐to‐moderate risk of bias, with particular strengths in controlling confounding factors and participant selection. Some studies showed medium risk due to missing data or inconsistencies in outcome reporting. Despite variability in outcome measures, the overall quality of evidence was deemed sufficient to support the conclusions of this systematic review. This systematic review was registered in PROSPERO (ID: CRD420251106164). An additional manual literature update was performed before manuscript submission to include recently published relevant studies.

## 3. Results

### 3.1. Characteristics of Included Articles

Figure 2 shows the flow diagram of a systematic review carried out using the PRISMA reporting criteria. The diagram describes the search strategy, inclusion, and exclusion of publications at each stage of detection.

A total of 493 publications were identified in three databases, including PubMed (105), Web of Science (157), and Scopus (231). After removal of 257 duplicates, 236 unique records remained for screening. The title and abstract analysis resulted in the exclusion of 173 articles because they did not fulfill inclusion criteria. The remaining 63 records were read, deleting 49 articles that were off‐topic. The evaluation includes a total of 14 publications for qualitative analysis.

### 3.2. Quality Assessment and Risk of Bias of Included Articles

The risk of bias for the included studies is summarized in Table 1.

**Table 1:** A tabular summary of the risk‐of‐bias assessment of the 14 studies.

Authors and Year	D1	D2	D3	D4	D5	Overall
Strauss et al. (2025) [63]	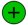	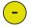	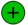	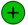	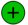	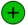
Segnini et al. (2021) [64]	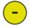	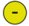	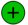	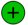	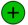	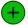
Hartlev et al. (2021) [3]	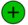	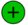	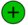	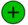	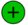	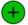
Basler et al. (2018) [65]	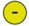	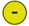	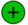	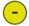	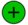	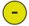
Cucchi et al. (2017) [66]	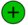	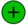	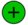	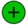	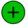	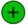
Solakoglu et al. (2019) [67]	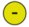	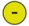	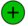	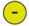	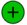	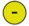
Wessing et al. (2017) [4]	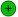	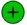	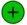	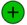	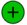	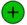
Jonker et al. (2021)[68]	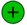	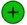	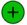	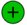	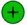	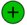
MacBeth et al, (2022) [16]	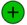	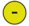	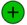	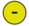	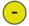	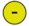
Scheyer et al. (2016) [6]	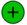	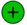	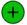	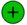	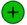	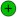
Shahdad et al. (2020) [69]	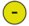	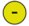	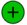	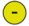	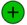	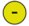
Arbab et al. (2016) [70]	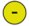	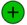	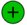	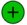	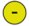	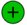
Santos et al. (2021) [29]	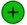	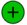	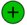	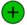	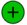	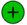
Cruz et al. (2021) [43]	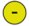	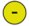	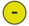	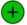	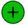	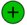

Most of the studies showed a low risk of bias due to confounding. Similarly, measurement bias and selection bias related to the participants were generally rated as low across the studies. Post‐exposure bias was also found to be low in the majority of cases. Conversely, bias due to missing data was classified as medium risk in several studies.

Outcome measurement bias could not be consistently assessed due to the heterogeneity of outcomes. Regarding reporting bias, more than half of the studies presented a low risk, while the remaining showed a medium risk. In conclusion, 10 studies were classified as having an overall low risk of bias, while four studies were assessed as having a medium risk of bias.

Table 2 summarizes the main features of the 14 randomized clinical trials included in this systematic review. These studies encompass a total of diverse patient samples, ranging from 16 to 75 individuals, with follow‐up periods varying widely between 3 and 32 months. The participants’ average ages spanned from early thirties to nearly 60 years, reflecting a broad adult population. The included studies assessed multiple clinical, radiographic, and histological parameters relevant to ARP and GBR. Among the investigated variables were buccal bone and soft tissue thickness, changes in ridge contour, implant survival and stability, as well as biological markers of inflammation and bone formation. Several studies also measured volumetric and linear dimensional changes, probing depths, bleeding indices, and postoperative complications such as membrane exposure or pain levels. This comprehensive array of outcomes allowed for a thorough comparison of different treatment modalities and materials. Collectively, these characteristics form the foundation for the qualitative synthesis and critical discussion that follow, providing a solid basis for evidence‐based recommendations.

**Table 2:** Variables studied.

Authors and year	Type of study	Study sample (in patients)	Follow‐up (months)	Age of sample (years)	Variables
Strauss et al. 2025 [63]	Randomized Controlled Trial	27	6	50.5 ± 19.7	Buccal bone thickness, buccal soft tissue thickness, contour changes, and measured at implant shoulder, 1, 2, and 3 mm.
Segnini et al. 2021 [64]	Randomized Controlled Clinical Trial	16	4	48.3	Type of soft tissue graft, tomographic parameters, clinical parameters, and histomorphometric composition. (bone, biomaterial, and soft tissue)
Hartlev et al. 2021 [3]	Randomized Controlled Clinical Trial	27	24	47.9	Implant survival, crown survival, probing depth, bleeding on probing, plaque index, marginal bone level, and soft tissue health.
Basler et al. 2018 [65]	Randomized Controlled Clinical Trial	23	32	56.6 ± 17.4	Type of membrane (resorbable vs. non‐resorbable), probing depth, bleeding on probing, plaque control record, radiographic bone levels, volumetric, and linear changes
Cucchi et al. 2017 [66]	Randomized Clinical Trial	40	12	52.4	Complication rate, implant stability, vertical bone gain, healing complications, membrane exposure, insertion torque, and bone defect size.
Solakoglu et al. 2019 [67]	Prospective Randomized Clinical Trial	20	5	Not explicitly stated	Type of graft, histomorphometry, inflammation markers, bone formation, and immunohistochemistry markers.
Wessing et al. 2017 [4]	Randomized Controlled Clinical Trial	49	6	38.6 ± 15.3	Defect height, membrane exposure, implant stability, and soft tissue healing.
Jonker et al. 2021 [68]	Randomized Controlled Clinical Trial	75	8	Not specified	Ridge width/resorption, soft tissue contour, pain, and profilometric evaluation post‐extraction.
MacBeth et al. 2022 [16]	Randomized, Single‐Blind Clinical Trial	43	4	32 ± 9.6	Ridge width, socket area changes, implant feasibility, and complications.
Scheyer et al. 2016 [6]	Randomized, Controlled, Multicenter Clinical Trial	40	6	18–70	Horizontal/vertical ridge preservation, wound closure, soft tissue inflammation, histomorphometry, and implant feasibility.
Shahdad et al. 2020 [69]	Randomized, controlled, clinical trial	30	6	40.1	Horizontal and vertical bone loss and need for further augmentation at implant.
Arbab et al. 2016 [70]	Randomized, controlled, blinded clinical trial	24	4	53 ± 15	Horizontal ridge width, vertical ridge height, vital bone, and trabecular space.
Santos et al. 2021 [29]	Randomized clinical trial	52	18	56.8 ± 12.3	Primary and secondary implant stability, pain, radiodensity, and peri‐implant conditions.
Cruz et al. 2021 [43]	Randomized clinical trial	26	3	43.6 ± 4.93	Horizontal/vertical bone changes, soft tissue healing index, pain, and analgesic use.

### 3.3. Risk of Bias Assessment

The risk of bias across the included randomized controlled trials was assessed considering key methodological domains such as the randomization process, deviations from intended interventions, missing data, outcome measurement, and selective reporting. Most studies demonstrated a low‐to‐moderate risk of bias due to their randomized design, adequate reporting of inclusion criteria, and use of standardized radiographic and clinical outcome measures. While most trials maintained consistent follow‐up periods, variability in intervention protocols (e.g., graft type, membrane type, and surgical technique) and measurement approaches (e.g., STL scans vs. linear probing, or variable radiographic reference points) could contribute to heterogeneity and measurement bias. The attrition bias was low across studies, with complete or near‐complete follow‐up reported in the majority of cases.

• Strauss et al. [63]—Low risk.
  The study demonstrated appropriate random sequence generation with adequate allocation concealment, ensuring balanced baseline characteristics (D1). The intervention protocol was clearly defined; however, limited reporting on adherence monitoring introduced minor concerns regarding deviations from the intended interventions (D2). Outcome data were complete, with no evidence of attrition bias (D3). Outcome assessment relied on standardized and objective clinical and radiographic parameters, minimizing measurement bias (D4). All prespecified outcomes were reported, with no indication of selective reporting (D5).
• Segnini et al. [64]—Some concerns.
  Although randomization was reported, insufficient details regarding allocation concealment raised potential bias in the randomization process (D1). The absence of clear blinding procedures suggested possible deviations from the intended interventions (D2). Outcome data were largely complete (D3), but histomorphometric evaluations may have introduced variability in the outcome measurement (D4). No evidence of selective reporting was observed (D5).
• Hartlev et al. [3]—Low risk of bias
  The study reported a well‐defined randomization process with adequate allocation concealment (D1) and strict adherence to intervention protocols (D2). Outcome data were complete with no significant loss to follow‐up (D3). The use of validated and objective outcome measures ensured a low risk of measurement bias (D4), and all predefined outcomes were consistently reported (D5).
• Basler et al. [65]—Some concerns.
  Randomization procedures were mentioned but not sufficiently detailed, raising concerns regarding allocation concealment (D1). The lack of information on blinding introduced potential bias due to deviations from the intended interventions (D2). Outcome data were complete (D3); however, heterogeneity in follow‐up and assessment methods raised concerns in the outcome measurement (D4). Reporting bias was not evident (D5).
• Cucchi et al. [66]—Low risk of bias.
  Adequate randomization and allocation concealment were described (D1), and intervention protocols were consistently applied (D2). Outcome data were complete (D3), and outcome measurements were standardized and objective (D4). No evidence of selective reporting was identified (D5).
• Solakoglu et al. [67]—Some concerns.
  Limited reporting on the randomization process raised concerns (D1), while variability in surgical procedures suggested possible deviations from the intended interventions (D2). Outcome data were complete (D3), but the lack of standardization in outcome assessment introduced potential measurement bias (D4). Reporting bias was not evident (D5).
• Wessing et al. [4]—Low risk of bias.
  The study demonstrated appropriate randomization and allocation procedures (D1), with no reported deviations from the intended interventions (D2). Outcome data were complete (D3), and measurements were standardized and reliable (D4). All outcomes were transparently reported (D5).
• Jonker et al. [68]—Low risk of bias.
  Robust randomization and allocation concealment were reported (D1), with high adherence to intervention protocols (D2). Outcome data were complete (D3), and validated clinical and patient‐reported outcomes minimized measurement bias (D4). No selective reporting was observed (D5).
• MacBeth et al. [16]—Some concerns.
  Randomization was adequately performed (D1), but the single‐blind design introduced potential bias due to deviations from the intended interventions (D2). Outcome data were complete (D3), while the lack of blinding may have influenced outcome measurement (D4). Selective reporting could not be entirely excluded (D5).
• Scheyer et al. [6]—Low risk of bias.
  The multicenter randomized design ensured appropriate randomization and allocation concealment (D1) with standardized protocols across centers (D2). Outcome data were complete (D3), and objective outcome measures were used (D4). All prespecified outcomes were reported (D5).
• Shahdad et al. [69]—Some concerns.
  Allocation concealment was not clearly described despite reported randomization (D1). Limited information on blinding raised concerns regarding deviations from the intended interventions (D2). Outcome data were complete (D3), but variability in outcome assessment methods introduced potential measurement bias (D4). Reporting bias was not evident (D5).
• Arbab et al. [70]—Some concerns.
  Insufficient reporting of the randomization process and allocation concealment raised concerns (D1). Limited information on adherence to intervention protocols suggested possible deviations (D2), while a small sample size increased the potential impact of missing data (D3). Outcome assessment methods were not fully standardized (D4), although no selective reporting was evident (D5).
• Santos et al. [29]—Low risk of bias.
  The study demonstrated appropriate randomization and allocation concealment (D1) with well‐controlled intervention protocols (D2). Outcome data were complete (D3), and standardized radiographic assessments minimized measurement bias (D4). No selective reporting was observed (D5).
• Cruz et al. [43]—Some concerns.
  Allocation concealment was not clearly reported (D1), and short follow‐up raised concerns regarding deviations from the intended interventions (D2). Outcome data were largely complete (D3), but subjective outcome measures introduced a potential bias in outcome assessment (D4). Reporting bias was not evident (D5).

## 4. Discussion

### 4.1. Overview of Membrane Types and Their Clinical Impact

The selection of a suitable membrane is a critical factor influencing the success of both ARP and GBR. Among the studies reviewed, resorbable collagen membranes (RCMs) were consistently favored for their biocompatibility, ability to support cellular ingrowth, and absence of a second surgery for removal [71–74]. For example, Study 1 demonstrated that RCMs combined with xenografts facilitated significant bone regeneration without complications, while Study 2 confirmed comparable results with RCMs in extraction sockets, leading to predictable ridge dimension maintenance [75–79]. Conversely, non‐resorbable membranes such as titanium‐reinforced PTFE used in Study 3 showed excellent space maintenance and bone volume outcomes but were associated with higher complication rates, particularly membrane exposure. This trade‐off between regenerative capacity and risk profile was echoed in Study 4, where the authors emphasized the importance of case selection when opting for a nonresorbable barrier.

The use of nonresorbable membranes represents a well‐established approach in GBR due to their superior space‐maintaining capacity and mechanical stability. However, their clinical success is highly dependent on careful case selection as complication rates remain relatively high [66]. This complication is not a minor event; rather, it has profound implications for treatment outcomes as early exposure predisposes to bacterial colonization, often necessitates premature removal of the barrier, and ultimately compromises the predictability of bone regeneration [80].

Soft tissue phenotype has emerged as a critical determinant of success in such cases. Thin tissues provide reduced mechanical protection against irritation from the membrane and present limited vascularization, both of which impair wound stability and healing potential. For this reason, a thin biotype is now regarded as a relative contraindication for the use of rigid, nonresorbable membranes in GBR procedures [81].

In addition to soft tissue characteristics, bone‐related parameters play a decisive role in treatment planning. Similarly, the geometry of the defect—especially the number of remaining bony walls—was shown to critically influence the regenerative potential [82]. Multi‐wall defects offer inherent mechanical support and vascular supply, thereby enhancing regenerative predictability, while single‐wall defects continue to pose considerable challenges, even when rigid membranes are employed.

Taken together, these findings emphasize that the indication for nonresorbable membranes should not rely solely on defect size or surgeon preference [83]. Instead, a multifactorial preoperative assessment, encompassing soft tissue thickness, bone density, and defect configuration, is required to minimize complications and optimize regenerative outcomes. Integrating predictive algorithms into clinical decision‐making may further enhance case selection, contributing to greater standardization and improved predictability in GBR protocols involving non‐resorbable barriers [84].

Notably, Study 5 explored the use of cross‐linked collagen membranes, highlighting prolonged resorption times and greater structural integrity, particularly advantageous in larger defects or simultaneous implant placement scenarios [66].

### 4.2. Graft Material Selection and Dimensional Stability

The type of graft material used in combination with membranes significantly influences both horizontal and vertical bone regeneration. Autografts, as reviewed in Study 6, offer superior osteoinductive and osteogenic properties but are limited by donor site morbidity and volume constraints [85–89]. On the other hand, xenografts—especially bovine‐derived—used in Studies 1, 2, and 7 provided excellent dimensional stability with low resorption rates, making them suitable for ARP when volume maintenance is a priority. Study 8 compared xenografts and allografts in socket preservation, finding that xenografts yielded more favorable outcomes in maintaining ridge width, although allografts exhibited faster resorption and earlier integration [67]. Synthetic bone substitutes were evaluated in Study 9, where beta‐tricalcium phosphate (β‐TCP) was associated with higher resorption rates and lower residual graft volume, suggesting that its use may be more appropriate in cases where rapid bone turnover is desired [90–93]. The synergistic effect of combining grafts with membranes was particularly evident in Study 10, which demonstrated that the application of a resorbable membrane significantly improved the integration and containment of the graft material [94–96].

### 4.3. Soft Tissue Considerations and Their Role in GBR Success

Soft tissue management is increasingly recognized as a cornerstone of GBR and ARP success. Study 11 highlighted that tension‐free primary closure over the membrane correlates with lower exposure rates and enhanced graft stability [64, 67]. Moreover, Study 12 explored the use of CTGs in combination with GBR, demonstrating improved soft tissue thickness, better esthetic outcomes, and reduced membrane exposure [63, 97, 98]. The impact of keratinized tissue width (KTW) was discussed in Study 13, where sites with adequate KTW exhibited better wound healing and less crestal bone loss post‐regeneration. Additionally, the type of flap design—full‐thickness versus split‐thickness—was investigated in Study 14, revealing that split‐thickness flaps might preserve more vascularity and reduce postoperative discomfort, albeit requiring more surgical expertise. These findings underscore the need to integrate both hard and soft tissue management strategies for long‐term stability and function [99–101].

### 4.4. Comparative Efficacy in Immediate vs. Delayed Implant Placement

Several studies provided insights into the timing of implant placement and its influence on regeneration outcomes [102–106]. Studies 6 and 7 compared immediate versus delayed implant placement in sites treated with GBR, concluding that while both approaches can be successful, delayed placement often results in greater bone fill and fewer complications [16, 107–109]. Study 2 emphasized that when ARP is properly performed using xenografts and RCMs, the dimensional changes are minimized enough to allow delayed implant placement without additional grafting. Study 9 also observed that immediate implants might benefit from more stable graft materials and rigid membranes to counteract the natural remodeling process post‐extraction [110]. These comparative analyses support a personalized approach, where timing is adapted based on patient anatomy, risk factors, and esthetic demands.

The determination of surgical timing in implant therapy is a multifactorial process influenced by anatomical, systemic, and esthetic considerations. Anatomical limitations often represent the primary determinant, particularly in cases where proximity to vital structures poses a risk. Cone‐beam computed tomography (CBCT) analysis identified that a residual bone height of less than 2 mm beneath the maxillary sinus significantly restricts the feasibility of immediate implant placement, necessitating either a staged sinus augmentation or a delayed approach [16].

Systemic health also exerts a critical influence on the decision‐making process and patients with uncontrolled diabetes, reflected by glycated hemoglobin (HbA1c) levels greater than 7%, demonstrated impaired healing and increased susceptibility to infection when subjected to immediate protocols. Consequently, in such cases, a delayed implant placement strategy has been advocated, providing sufficient time for metabolic control and thereby reducing the risk of peri‐implant complications [44].

In addition to anatomical and systemic factors, esthetic demands significantly shape the timing of surgical intervention. This finding underscores the importance of preserving peri‐implant mucosal architecture and highlights the role of surgical timing in optimizing esthetic outcomes, particularly in the anterior maxilla [111]. By contrast, for medically compromised cohorts, delayed protocols remain more favorable, ensuring greater predictability and minimizing risks related to impaired healing capacity [112].

Overall, the available evidence indicates that the choice of surgical timing should not be standardized but rather individualized through a comprehensive assessment of anatomical, systemic, and esthetic parameters [113].Incorporating advanced imaging modalities such as CBCT, monitoring systemic risk factors including glycemic control, and accounting for patient‐specific esthetic expectations allows clinicians to tailor treatment protocols. This patient‐centered approach contributes to improved safety, enhanced esthetic integration, and long‐term stability of implant‐supported rehabilitations [114].

### 4.5. Toward a Personalized, Evidence‐Based Protocol

The cumulative evidence from the 14 studies highlights the importance of tailoring regenerative protocols based on defect morphology, patient‐related factors, and clinical priorities.

Patients‐related factors further influence outcomes [115]. Smoking, particularly ≥10 cigaretes/day, has been consistently associated with impaired healing, higher graft resorption, and increased complications [116].

Clinical priorities also guide treatment planning. In medically fragile or time‐sensitive cases, single‐stage procedures reduce chair time without compromising outcomes [117]. Where economic constraints are relevant, cost‐effective biomaterials such as collagen membranes remain a reliable option due to their favorable handling and resorption characteristics [118].

Collectively, these factors underscore the need for an individualized, evidence‐based approach to grafting protocols, integrating anatomical, behavioral, and practical considerations to optimize long‐term success [119].

For instance, patients with thin biotypes or compromised soft tissue may benefit more from CTG use, as seen in Study 12, while those requiring vertical augmentation may be better candidates for nonresorbable membranes with rigid support, as indicated in Studies 3 and 4. The predictability of xenograft and RCM combinations across multiple studies (1, 2, 7, and 8) supports their use as a standard ARP protocol [120]. Furthermore, clinician expertise and flap management techniques (Study 14) play a decisive role in outcome predictability. Flap management plays a critical role in the success of regenerative procedures as it directly affects vascularization, wound stability, and overall healing potential [121].

In recent years, adjunctive innovations have further improved clinical predictability, particularly in challenging scenarios involving severe ridge deficiencies [122]. Er:YAG laser–assisted healing has demonstrated favorable outcomes by reducing bacterial contamination and enhancing soft tissue biocompatibility [123]. Likewise, the incorporation of platelet‐rich fibrin (PRF) into regenerative protocols has gained substantial attention. Multiple studies have documented its ability to promote angiogenesis, accelerate soft tissue maturation, and improve graft integration, offering a biologically driven adjunct in cases of advanced horizontal bone loss) [124].

Overall, current evidence suggests that successful regenerative outcomes depend not only on the choice of biomaterials but also on precise soft tissue management and the integration of adjunctive technologies. A systematic evaluation of flap design, closure technique, and biologically active adjuncts is therefore essential to optimize vascularization, enhance predictability, and improve long‐term clinical success. Future protocols should increasingly integrate digital planning, patient‐specific anatomical assessments, and minimally invasive techniques, aligning regenerative procedures with broader goals of personalized care and implant prosthetic planning [125].

### 4.6. Limitations

A limitation of the present review is that most of the included studies did not clearly specify whether the evaluated extraction sites were located in the anterior or posterior regions. Consequently, subgroup analysis based on extraction site location was not feasible. Considering that post‐extraction ridge remodeling may differ between anterior and posterior areas, particularly in the anterior maxilla, where greater dimensional changes are commonly observed, future clinical studies should report this information more consistently to improve the applicability and interpretation of the available evidence.

## 5. Conclusion

The current literature outlines a progressively refined and patient‐centered paradigm in the management of ARP and GBR. Rather than prescribing universal solutions, the evidence supports a stratified approach, wherein clinical decision‐making is shaped by the interplay between anatomical complexity, biological responsiveness, and individual patient profiles. Within this framework, therapeutic choices are no longer driven solely by material properties or procedural efficacy but by a broader assessment that includes soft tissue dynamics, surgical timing, and systemic considerations.

Notably, the integration of soft tissue management into regenerative protocols has transitioned from an ancillary role to a fundamental determinant of long‐term stability. Likewise, the nuanced selection of grafting materials—balancing osteoinductive potential, structural integrity, and immunologic compatibility—reflects a shift toward precision‐driven regenerative strategies.

Despite the methodological rigor of the analyzed studies, the limited inclusion of complex clinical scenarios and the absence of long‐term, multicenter data represent critical areas for further investigation.

Although the included studies were conducted with considerable methodological rigor, their scope often remains confined to relatively controlled and simplified conditions.

One of the main limitations is the restricted consideration of complex clinical situations that are frequently encountered in daily practice.

Such scenarios, which involve patients with systemic comorbidities, anatomical challenges, or multifactorial risk factors, are often excluded from experimental protocols.

This selective inclusion may reduce the external validity of the findings, making it difficult to generalize the results to broader populations.

Furthermore, the absence of long‐term follow‐up data limits the capacity to evaluate the stability and predictability of the reported outcomes over time.

Short‐ and medium‐term results, while encouraging, do not necessarily reflect the performance of the interventions over the course of several years.

In addition, the lack of multicenter trials restricts the possibility of assessing reproducibility across diverse clinical settings and professional expertise.

Evidence generated from single‐center studies, though valuable, may be influenced by local protocols, operator skill, and patient selection biases.

This underlines the importance of conducting large‐scale investigations that integrate heterogeneous samples and varied treatment contexts.

Overall, future research should aim to overcome these gaps in order to provide more comprehensive, reliable, and clinically applicable conclusions. Moreover, the lack of economic evaluations underscores the necessity of aligning clinical outcomes with cost effectiveness, particularly in resource‐constrained settings. Cost effectiveness is a crucial factor in regenerative dentistry, particularly in resource‐limited settings.

Autogenous grafts remain a predictable and economical option, eliminating material costs while providing osteogenic properties. Sterilization methods also influence cost and feasibility: simplified approaches, such as chlorhexidine disinfection, have shown outcomes comparable to those of autoclaving under controlled conditions, making them suitable alternatives where advanced equipment is unavailable.

Membrane selection similarly reflects the balance between efficacy and affordability. It is known that adherence to surgical and infection control standards may be more critical to clinical success than the intrinsic cost of the biomaterial.

Overall, the evidence indicates that predictable regenerative outcomes can be achieved even in low‐resource environments by integrating autograft use, simplified sterilization, and affordable membrane alternatives, thereby expanding accessibility without compromising the success rates.

Future research should prioritize extended follow‐up, the inclusion of high‐risk patient cohorts, and integration of translational innovations such as bioresponsive scaffolds and biomarker‐driven protocols.

Bioresponsive scaffolds are emerging as promising tools in regenerative dentistry because of their ability to adapt to the local biological environment [126]. One example is represented by pH‐sensitive hydrogels, which regulate their degradation in response to inflammatory conditions. Experimental studies in animal models have shown that these scaffolds accelerate resorption in acidic environments (pH below 5.5), ensuring a controlled release of bioactive factors and preventing the accumulation of unwanted degradation products. These properties are particularly relevant in oral tissues, where inflammation frequently alters the microenvirnment and compromises the stability of traditional scaffolds.

In clinical settings, biomarker‐guided protocols provide additional benefits.

Detecting these markers at an early stage enables timely intervention and reduces the likelihood of disease progression.

Overall, both bioresponsive scaffolds and biomarker‐based monitoring represent a shift toward more personalized regenerative approaches, improving predictability and supporting long‐term implant stability. Ultimately, the success of regenerative therapy lies not in the standardization of techniques but in the clinician’s ability to synthesize scientific evidence with individual patient needs, thereby achieving a personalized and sustainable therapeutic outcome.

Personalization in regenerative dentistry is increasingly supported by both biological and digital innovations. Genetic profiling, such as the analysis of bone morphogenetic protein 2 polymorphisms through polymerase chain reaction testing, allows clinicians to anticipate patient‐specific variations in bone healing and tailor regenerative protocols accordingly. At the same time, digital workflows are improving precision in surgical planning. Artificial intelligence–based ridge analysis enables more accurate assessment of bone morphology and supports individualized treatment strategies.

Sustainability also plays a growing role in contemporary practice. The use of recyclable titanium meshes provides mechanical stability while reducing environmental impact. Similarly, streamlined surgical kits designed to minimize disposable instruments have been shown to reduce clinical waste and cut material costs by ~37%.

Overall, combining patient‐specific diagnostics with environmentally conscious protocols represents a step toward more precise, cost‐effective, and sustainable regenerative dentistry, aligning clinical innovation with broader healthcaree priorities.

**Nomenclature**
ARP: Alveolar ridge preservation
β‐TCP: beta‐tricalcium phosphate
CTG: connective tissue graft
GBR: guided bone regeneration
KTW: keratinized tissue width
PTFE: polytetrafluoroethylene
RCM: resorbable collagen membrane.

## Author Contributions

**Angelo Michele Inchingolo**: conceptualization, methodology, writing – original draft. **Grazia Marinelli**: data curation, formal analysis, visualization. **Pietro Lauria**: investigation, resources, validation. **Pierluigi Marotti**: formal analysis, data visualization. **Silvia Chieppa**: writing – review and editing, project administration. **Francesco Inchingolo**: supervision, methodology, writing – review and editing. **Andrea Palermo**: validation, resources, writing – review and editing. **Massimo del Fabbro**: methodology, supervision, formal analysis. **Alessio Danilo Inchingolo**: investigation, data curation, writing – original draft. **Gianna Dipalma**: conceptualization, writing – review and editing, project administration.

## Funding

No funding was received for this manuscript. Open access publishing facilitated by Universita degli Studi di Bari Aldo Moro, as part of the Wiley ‐ CRUI‐CARE agreement.

## Conflicts of Interest

The authors declare no conflicts of interest.

## Data Availability

Data sharing not applicable to this article as no datasets were generated or analyzed during the current study.
